# Budesonide and Calcitriol Synergistically Inhibit Airway Remodeling in Asthmatic Mice

**DOI:** 10.1155/2018/5259240

**Published:** 2018-05-02

**Authors:** Jun Qian, Yaqin Xu, Zhiwei Yu

**Affiliations:** Department of Pediatrics, Wuxi Children's Hospital Affiliated to Nanjing Medical University, Wuxi, Jiangsu, China

## Abstract

**Background and Objective:**

While calcitriol can inhibit airway remodeling in asthmatic mice, the mechanism remains unclear. The purpose of this study was to explore the mechanism of action of calcitriol on airway remodeling in asthma and its interaction with budesonide.

**Methods:**

A mouse model of asthma was established by allergic sensitization and challenge with ovalbumin. The mice were treated with budesonide, calcitriol, or budesonide plus calcitriol. The expression of airway remodeling-related proteins, transforming growth factor *β* (TGF*β*) signaling pathway-related proteins, the glucocorticoid receptor, and vitamin D receptor (VDR) was determined by immunohistochemical staining and Western blot analysis. Quantitative real-time PCR was used to determine the expression of microRNA-21 (miR-21) in the lung tissue of mice.

**Results:**

Monotherapy with budesonide or calcitriol inhibited the high expression of collagen type I protein and upregulated the low expression of Smad7 in asthmatic mice. There was a synergistic interaction between budesonide and calcitriol in combined treatment. The expression of miR-21 in the combined treatment group was significantly lower than that in the calcitriol treatment group. VDR expression in the combined treatment group was significantly higher than that of the calcitriol treatment group.

**Conclusion:**

Budesonide and calcitriol have a synergistic effect on airway remodeling in asthmatic mice.

## 1. Introduction

Bronchial asthma is a heterogeneous disease with complex pathogenesis characterized by chronic airway inflammation and airway hyperresponsiveness. Airway remodeling in asthma is closely associated with persistent and severe asthmatic symptoms and airway hyperresponsiveness. Moreover, TGF*β* plays an important role in the pathogenesis of airway remodeling in bronchial asthma [1]. The TGF*β*/Smad signaling pathway is important for the biological functioning of TGF*β* [2]. The imbalanced miR-21 expression is related to fibrosis and the TGF*β*/Smad signaling pathway [3]. Budesonide is a commonly used inhaled corticosteroid in asthmatic patients and has an anti-inflammatory effect on asthma via binding to the glucocorticoid receptor (GR). However, glucocorticoid therapy cannot reduce the high expression of TGF*β* in asthmatic patients, suggesting that glucocorticoid therapy alone cannot inhibit the factors involved in airway remodeling [4]. Vitamin D deficiency is associated with allergic diseases (e.g., asthma) in children. Calcitriol, the active metabolite of vitamin D3, can bind to both the VDR and retinoid X receptor (RXR) in sequence to form a heterodimer [5, 6]. The monotherapy with calcitriol or budesonide can inhibit airway remodeling in asthma [7, 8], but whether a combination of both exhibits a synergistic or additive effect remains unclear. In this *in vivo* study, we established a model of asthma in BALB/c mice by ovalbumin (Ova) sensitization and challenge and then applied calcitriol, budesonide, or a combination of both to investigate the effect of calcitriol on airway remodeling in asthma, and its interaction with budesonide.

## 2. Materials and Methods

### 2.1. Animals

Female BALB/c mice were obtained from the Experimental Animal Center of Nanjing University. Mice were aged 6 to 8 weeks and housed in plastic cages (8 animals per cage) in an air-conditioned room at 23°C with a 12-hour light-dark cycle. Food and tap water were available ad libitum. All animals were kept under conventional pathogen-free conditions at the Nanjing Medical University Research Animal Facility, and all procedures were obeyed in accordance with Nanjing Medical University guidelines.

### 2.2. Experiment Protocol

A total of 40 female BALB/c mice were randomly divided into five groups (*n*=8): (1) normal control group, (2) asthma model group, (3) budesonide treatment group, (4) calcitriol treatment group, and (5) combined treatment group. All BALB/c mice, except those in the normal control group, were intraperitoneally injected with 50 *μ*g Ova (Sigma-Aldrich, St. Louis, MO, USA) dissolved in phosphate-buffered saline (PBS) and a 0.2 mL suspension containing 1 mg aluminum hydroxide for sensitization on days 1 and 14. As a challenge, beginning on day 21, the mice inhaled a 5 mL solution containing 1% ovalbumin over 30 min, once daily, for eight weeks to establish a mouse model of asthma [8, 9]. In the budesonide treatment group, 0.5 h before the Ova challenge, the mice inhaled 5 mL PBS with 1 mg budesonide (AstraZeneca Pty Ltd., New South Wales, Australia) dissolved by a nebulizer over 30 min [8]. In the calcitriol treatment group, 1 h before Ova challenge, the mice were intraperitoneally injected with 100 ng calcitriol (Sigma-Aldrich, St. Louis, MO, USA) dissolved in 0.2 mL PBS [7]. In the combined treatment group, the mice were administered a budesonide inhalation and intraperitoneal injection of calcitriol as in the budesonide treatment and calcitriol treatment groups. The mice in the normal control group were intraperitoneally injected with PBS for sensitization and given a PBS inhalation for the challenge. Twenty-four hours after the last aerosol exposure, mice were sacrificed by intraperitoneal injection of sodium pentobarbital (100 mg/kg).

Lung tissues were collected for histopathological staining, immunohistochemistry assessment, Western blot analysis, and quantitative real-time PCR (qRT-PCR) examination. Paraffin-embedded sections (5 *μ*m) were stained with hematoxylin and eosin (H&E) for assessment of eosinophil infiltration, with periodic acid-Schiff (PAS) for assessment of mucus secretion, and with Van Gieson (VG) for assessment of collagen deposition. Expressions of collagen type I (COL I), collagen type III (COL III), *α*-smooth muscle actin (*α*-SMA), fibronectin (FN), TGF*β*1, TGF*β* type I receptor (TGF*β*RI), TGF*β* type II receptor (TGF*β*RII), Smad2, Smad3, phosphorylated Smad2 (pSmad2), phosphorylated Smad3 (pSmad3), Smad4, Smad7, GR, VDR, RXR, and GAPDH (Abcam, Cambridge, MA, USA) in the lung tissues were demonstrated using Western blot analysis and immunochemistry technique according to the manufacturer's instructions as previously described [8]. The expression of miR-21 was measured by qRT-PCR and normalized to U6 small noncoding RNA. qRT-PCR was performed using SYBR® Premix Ex TaqTM II Kit (TaKaRa Biotechnology Co., Ltd., Dalian, China) on an ABI PRISM 7900-HT Sequence Detection System (Applied Biosystems, Foster City, CA, USA) according to the manufacturer's instructions. The primers used for qRT-PCR in this study are summarized in Table 1.

### 2.3. Statistical Analysis

Data were expressed as mean ± SD. Results were analyzed using analysis of variance (ANOVA). All post hoc comparisons were carried out using the Tukey test for significant effects. To investigate the presence or absence of an interaction between budesonide and calcitriol, the 2 × 2 factorial experimental design was used. *P* < 0.05 was considered to be significant.

## 3. Results

### 3.1. Effect of Budesonide and Calcitriol on Airway Inflammation and Remodeling in Asthmatic Mice

**Figure 1:**
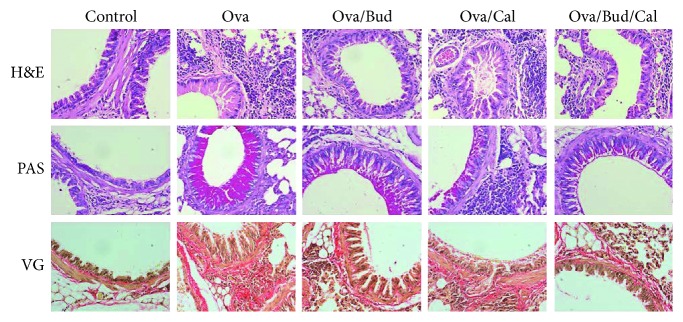
Effect of budesonide (Bud) and calcitriol (Cal) on airway inflammation and remodeling in ovalbumin- (Ova-) treated mice. H&E: airway eosinophilic infiltration in hematoxylin and eosin-stained sections. PAS: airway mucus secretion in periodic acid-Schiff-stained samples. VG: airway collagen proliferation in Van Gieson-stained sections (original magnification ×400).

Compared with the control mice, asthmatic mice exhibited a series of pathological changes, including enhanced airway eosinophil infiltration, increased airway mucus secretion, and excessive collagen proliferation around the airway. Such changes were alleviated after the administration of budesonide, calcitriol, or both (Figure 1).

### 3.2. Effect of Budesonide and Calcitriol on the Expression of Airway Remodeling-Related Proteins in Asthmatic Mice

**Figure 2:**
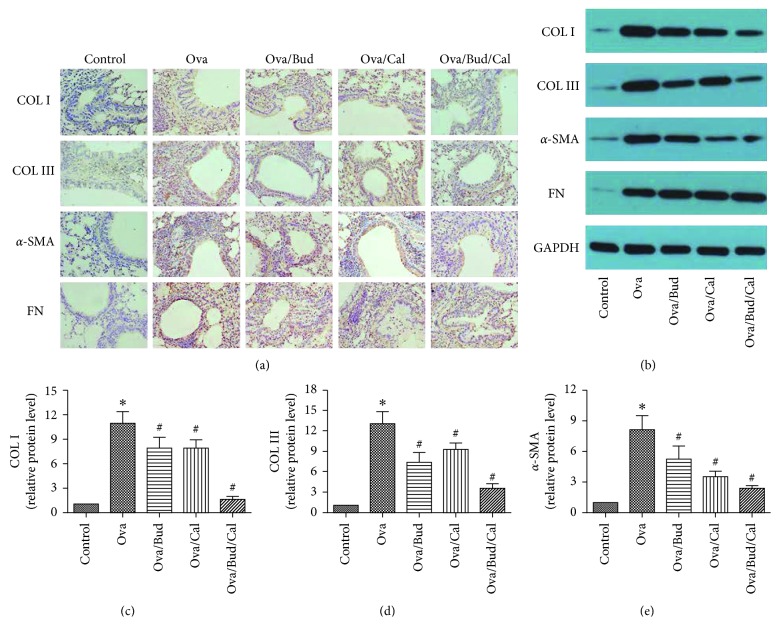
Effect of budesonide (Bud) and calcitriol (Cal) on the expression of airway remodeling-related proteins in ovalbumin- (Ova-) treated mice. (a) The lung tissue of the mice was fixed, embedded, and sectioned for immunohistochemical staining (DAB stain, original magnification ×400). (b) The total protein was extracted from the lung tissue of mice for Western blot analysis. (c, d, e) The expression of COL I, COL III, and *α*-SMA in the lung tissue of mice was determined by Western blot analysis, with GAPDH as an internal reference (^∗^*P* < 0.05 versus control group, ^#^*P* < 0.05 versus Ova-treated group).

Compared with the control mice, asthmatic mice showed elevated expression of airway remodeling-related proteins (i.e., COL I, COL III, *α*-SMA, and FN). Following drug treatment (e.g., budesonide, calcitriol, or both), the expression of COL I, COL III, and *α*-SMA was inhibited; however, there was no change in FN expression in the lung tissue (Figures 2 and 2). The results revealed that budesonide or calcitriol monotherapy could inhibit the high expression of COL I, COL III, and *α*-SMA in Ova-treated mice (Figures 2–2), there was an interaction between budesonide and calcitriol (*F*=6.08,  *P* < 0.05), and the interaction between budesonide and calcitriol was synergistic (*t*=2.83,  *P* < 0.05).

### 3.3. Effect of Budesonide and Calcitriol on the Expression of the TGFβ/Smad Signaling Pathway-Related Proteins in Asthmatic Mice

**Figure 3:**
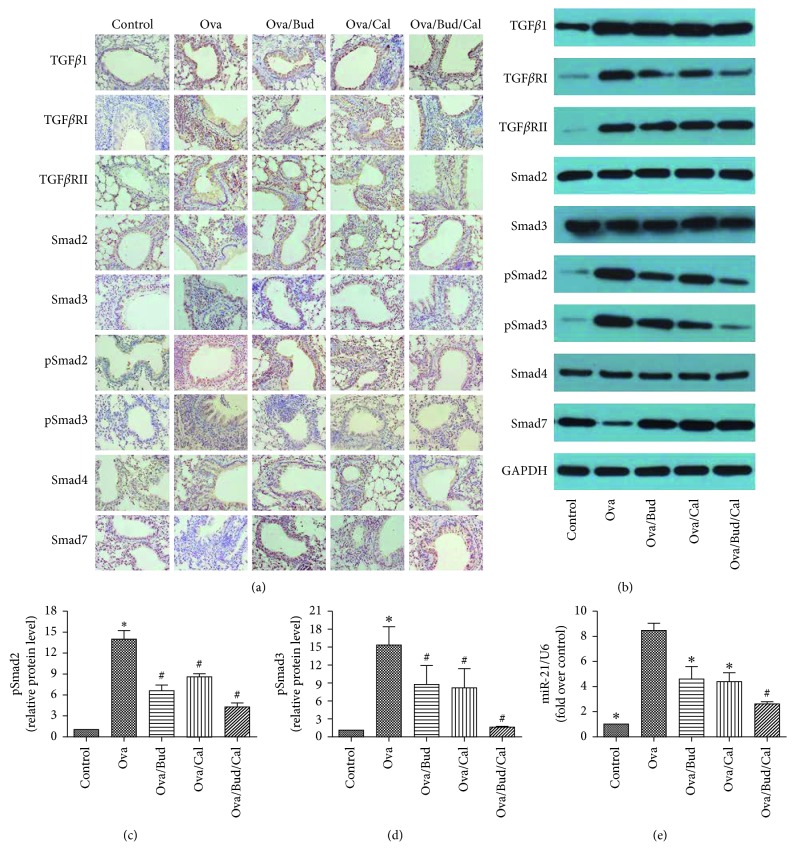
Effect of budesonide (Bud) and calcitriol (Cal) on the expression of the TGF*β*/Smad signaling pathway-related proteins and miR-21 in ovalbumin- (Ova-) treated mice. (a) The lung tissue of the mice was fixed, embedded, and sectioned for immunohistochemical staining (DAB stain, original magnification ×400). (b) The total protein was extracted from the lung tissue of mice for Western blot analysis. (c, d) The expression of pSmad2 and pSmad3 in the lung tissue of mice was determined by Western blot analysis, with GAPDH as an internal reference (^∗^*P* < 0.05 versus control group, ^#^*P* < 0.05 versus Ova-treated group). (e) miR-21 expression was determined by qRT-PCR with U6 as an internal reference after total RNA was extracted from the lung tissue of mice (^∗^*P* < 0.05 versus Ova-treated group, ^#^*P* < 0.05 versus Ova/Cal-treated group).

Compared with the control mice, asthmatic mice exhibited increased expression of TGF*β*1, TGF*β*RI, TGF*β*RII, pSmad2, and pSmad3, as well as decreased expression of Smad7; however, there were no significant changes in the expression of Smad2, Smad3, and Smad4. Following treatment with budesonide, calcitriol, or both, the expression of TGF*β*RI, pSmad2, and pSmad3 in the lung tissue of asthmatic mice was downregulated, while Smad7 expression was upregulated. However, there was no significant change in the expression of TGF*β*1 and TGF*β*RII after drug administration (Figures 3–3).

### 3.4. Effect of Budesonide and Calcitriol on miR-21 Expression in Asthmatic Mice

The expression of miR-21 in the lung tissue of asthmatic mice was significantly higher than that in control mice. After the administration of budesonide, calcitriol, or both, miR-21 expression was decreased. miR-21 expression in the combined treatment group was significantly lower than that in the calcitriol treatment group (Figure 3).

### 3.5. Effect of Budesonide and Calcitriol on the Expression of GR, VDR, and RXR in Asthmatic Mice

**Figure 4:**
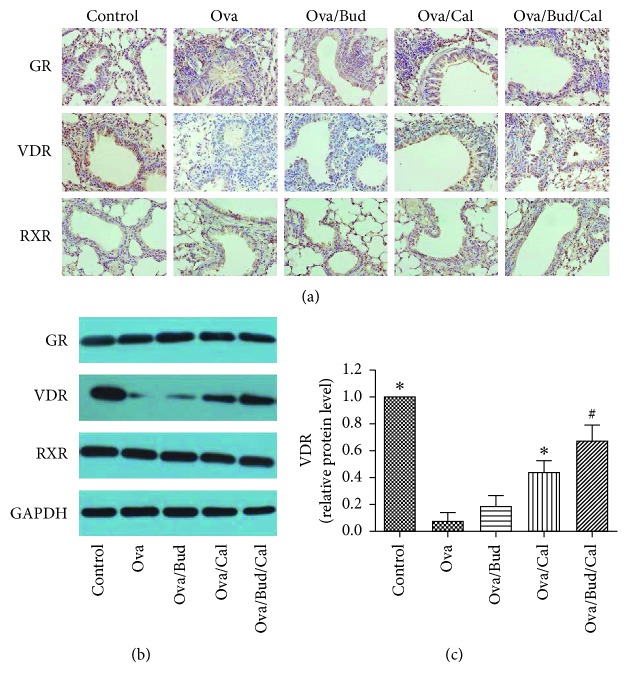
Effect of budesonide (Bud) and calcitriol (Cal) on the expression of GR, VDR, and RXR in ovalbumin- (Ova-) treated mice. (a) The lung tissue of the mice was fixed, embedded, and sectioned for immunohistochemical staining (DAB stain, original magnification ×400). (b) The total protein was extracted from the lung tissue of mice for Western blot analysis. (c) VDR protein expression in the lung tissue of mice was determined by Western blot analysis, with GAPDH as an internal reference (^∗^*P* < 0.05 versus Ova-treated group, ^#^*P* < 0.05 versus Ova/Cal-treated group).

VDR protein expression in the lung tissue of asthmatic mice was significantly lower than that of control mice. VDR expression was upregulated after the administration of calcitriol or budesonide combined with calcitriol. The expression of VDR protein in the combined treatment group was significantly higher than that in the calcitriol treatment group. The expression of GR and RXR in lung tissue revealed that there was no significant difference between these groups of mice (Figure 4).

## 4. Discussion

Although glucocorticoids are currently first-line drugs for asthma, the long-term inhalation or systemic administration of steroids will cause some endocrinal or metabolic adverse reactions that affect the growth and development of children. Moreover, chronic airway inflammation in asthma can cause airway remodeling, resulting in poor efficacy of glucocorticoid therapy in some patients with asthma [4]. It is necessary to identify a drug which, if combined with steroids, can produce a synergic effect to reduce the dosage and adverse effects of steroid treatment, or that can be used as an alternative to steroids for the treatment of pediatric asthma.

Vitamin D functions as a biologically active hormone analogue in the human body. Calcitriol, the highly active metabolite of vitamin D3, can maintain the balance of calcium and phosphorus metabolism and physiologically prevents rickets by acting on target organs (e.g., intestine, kidney, and bone). However, recent studies have shown that calcitriol is involved in cellular proliferation, differentiation, and immunomodulatory processes as a hormone analogue [10].

Calcitriol plays its immunomodulatory role via VDR, which is a member of the nuclear receptor family. After calcitriol binds to VDR, the conformation of VDR changes and then binds to the RXR to form a heterodimer, which enters the nucleus, binds to reactive elements of vitamin D, and promotes the transcription of vitamin D-related genes [11]. Vitamin D is associated with the pathogenesis of various diseases. In particular, a vitamin D deficiency can promote pulmonary fibrosis [12]. A mouse model of unilateral ureteral obstruction also demonstrated that the downregulation of VDR expression in renal tissue was associated with an epithelial mesenchymal transition and renal fibrosis [13]. By establishing a mouse model of asthma and calcitriol intervention, it was revealed that calcitriol can reduce airway remodeling in asthmatic mice by inhibiting the activation of nuclear factor *κ*B [14]. Asthmatic patients with low levels of vitamin D are prone to impaired pulmonary function and enhanced airway hyperresponsiveness, resulting in an increased dosage and adverse reactions in response to glucocorticoid therapy [15, 16].

Vitamin D can exert an anti-inflammatory effect through both Th2 and Th17 cells [17, 18], as well as the inhibition of airway eosinophilic inflammation in asthmatic mice [19]. In this study, we established a mouse model of asthma by ovalbumin sensitization and challenge and applied budesonide, calcitriol, or budesonide plus calcitriol to treat the asthmatic mice. Compared with control mice, the asthmatic mice exhibited pathological changes (e.g., airway inflammation and airway remodeling), which were alleviated following drug administration. This suggests that budesonide and calcitriol can prevent both airway inflammation and airway remodeling.

Airway remodeling in asthma refers to changes in the airway wall structure, including reversible and incompletely reversible airway changes. An early-stage asthma attack is characterized by an airway mucosal edema, airway infiltration of inflammatory cells (e.g., eosinophils), inflammatory cytokines produced by recruited cells, increased airway responsiveness, and limited airflow reversibility. In the early stages of asthmatic airway inflammation, elevated TGF*β*1 levels can promote tissue repair in an ideal microenvironment in response to airway damage induced by inflammation. However, in chronic airway inflammation due to repeated antigenic stimulation, persistently high levels of TGF*β*1 can cause tissue fibrosis and airway remodeling, exacerbating the anti-inflammatory response induced by TGF*β*1. Therefore, the airway wall thickens, and incomplete reversible airflow obstruction occurs [20].

The present study found that compared with the control mice, asthmatic mice had an increased expression of COL I, COL III, *α*-SMA, and FN in the lung tissue, as well as airway remodeling. Treatment with budesonide, calcitriol, or budesonide plus calcitriol inhibited the high expression of COL I, COL III, and *α*-SMA in the lung tissue of asthmatic mice; however, treatment had no influence on the elevated expression of FN protein, indicating that the chronic airway remodeling caused by long-term ovalbumin challenge in asthmatic mice was not completely reversible. This study demonstrated that budesonide or calcitriol monotherapy can inhibit activation of the TGF*β*/Smad signaling pathway in asthmatic mice, thereby inhibiting TGF*β*1-induced airway remodeling. Moreover, combination therapy with budesonide and calcitriol had a synergistic effect in inhibiting airway remodeling in asthmatic mice.

This study found that compared with the control mice, asthmatic mice exhibited decreased VDR expression in the lung tissue and calcitriol upregulated VDR expression to prevent airway remodeling. Combination therapy with budesonide and calcitriol further promoted the upregulation of VDR expression by calcitriol and exerted a synergistic effect in inhibiting airway remodeling; however, it presented no impact on the expression of GR and RXR in the airway. This finding suggests that the synergistic effect of calcitriol and budesonide in inhibiting airway remodeling is not achieved by interfering with GR expression in asthmatic mice.

Airway samples from healthy individuals and asthmatic patients were collected by fiberoptic bronchoscopy to determine GR mRNA and protein expression. In addition, the results revealed no significant difference between the healthy individuals and asthmatic patients [21]. A fiberoptic bronchoscopic biopsy of airway samples from asthmatic patients showed that there was no significant difference in GR mRNA and protein expression between the glucocorticoid and nonglucocorticoid therapy groups [22]. This suggests that the effects of glucocorticoids in asthma are not mediated by upregulating or downregulating GR expression in the airway. Glucocorticoids bind to GR to form a complex, which is subsequently translocated into the nucleus to bind to glucocorticoid response elements in the promoter region of the target gene or interact with transcription factors, thereby directly or indirectly regulating the transcription of the target gene. In the resting state, GR is distributed in the cytoplasm as receptor complexes. After GR binds to glucocorticoids, inhibitory chaperones (e.g., heat shock protein 90) in the complex changes its conformation and dissociates. The complex then enters the nucleus and binds to hormone response elements in the form of homodimers. Additionally, the complex recruits auxiliary activators, leading to chromatin remodeling and depolymerization, thereby regulating the transcriptional activity of the target gene and upregulating the expression of anti-inflammatory factors [23].

MicroRNAs (miRNAs) are a group of functional noncoding small RNAs consisting of 17–24 nucleotides, which inhibits target gene translation or degrades the target mRNA by binding to the 3′ noncoding region of target mRNA. In this manner, miRNAs can downregulate the expression of the target gene [9, 24]. In addition, miRNAs play an important role in fibrotic disease and attract attention as a novel target of gene therapy [25]. The pathogenesis of pulmonary fibrosis is related to an imbalance of miR-21 expression. TGF*β*1 stimulates human lung fibroblasts to increase the expression of miR-21 in human lung fibroblasts [26]. Both the bioinformatics analysis and dual luciferase reporter assay have shown that Smad7 and TGF*β*RII are target genes of miR-21 [27]. Our study demonstrated that budesonide and calcitriol can prevent airway remodeling by inhibiting miR-21 expression in the lung tissue of asthmatic mice, upregulating Smad7 protein expression, a target gene of miR-21. Moreover, there was no effect on the protein expression of TGF*β*RII, another target gene of miR-21. This suggests that TGF*β*RII is regulated not only by miR-21, and it may also involve other miRNAs; an miRNA can regulate the mRNAs of multiple target genes, and an mRNA can be simultaneously regulated by multiple miRNAs [28].

Our study showed that combination therapy with budesonide and calcitriol can promote the upregulation of VDR expression by calcitriol and downregulate miR-21 expression to increase Smad7 protein expression, a target gene of miR-21. This will induce a synergistic inhibitory effect on airway remodeling in asthmatic mice. Future clinical trials are required to evaluate the safety and efficacy of combination therapy with budesonide and calcitriol in asthmatic patients, which will provide references for the treatment of airway remodeling in asthma.

## Figures and Tables

**Table 1:** The primers used for quantitative real-time PCR.

RNA	Forward primer	Reverse primer
miR-21	GCGCTAGCTTATCAGACTGA	GTGCAGGGTCCGAGGT
U6	CTCGCTTCGGCAGCACATATAC	CGAATTTGCGTGTCATCCTTGC

## Data Availability

The data used to support the findings of this study are available from the corresponding author on reasonable request.
